# Comparing the Effects of Propofol and Thiopental on Human Renal HEK-293 Cells With a Focus on Reactive Oxygen Species (ROS) Production, Cytotoxicity, and Apoptosis: Insights Into Dose-Dependent Toxicity

**DOI:** 10.7759/cureus.74120

**Published:** 2024-11-20

**Authors:** Veli Fahri Pehlivan, Basak Pehlivan, Erdogan Duran, İsmail Koyuncu

**Affiliations:** 1 Anesthesiology, Harran University, Şanliurfa, TUR; 2 Anesthesiology and Reanimation, Harran University, Şanlıurfa, TUR; 3 Medical Biochemistry, Harran University, Şanliurfa, TUR; 4 Biochemistry, Harran University, Şanliurfa, TUR

**Keywords:** anesthesia, apoptosis, cytotoxicity, human kidney cells (hek-293), propofol-thiopental, reactive oxygen species (ros)

## Abstract

Objectives: Propofol and thiopental are widely used as hypnotic, sedative, antiepileptic, and analgesic agents in general anesthesia and intensive care; however, their side effects remain unknown. They are used for long periods and at high doses for sedation in total intravenous anesthesia (TIVA) and intensive care units. Long-term and high-dose use of these drugs can lead to accumulation in plasma and tissues, resulting in high drug concentrations and increasing the risk of potential toxicity (e.g., nephrotoxicity). In our study, the cytotoxic and apoptotic effects of propofol and thiopental on kidney cells (HEK-293) and their effects on the formation of reactive oxygen species (ROS) when used in high doses were investigated and compared in vitro.

Materials and methods: The half-maximal inhibitory concentration (IC_50_) of each drug in HEK-293 cells was determined using the 3-(4,5-dimethylthiazol-2-yl)-2,5-diphenyltetrazolium bromide method. The apoptotic effects were assessed at two different doses of each drug using the annexin V method. Morphological examinations were conducted using the acridine orange/ethidium bromide method, and intracellular ROS levels were determined by flow cytometry.

Results: The IC_50_ values of propofol and thiopental for HEK-293 cells were 206.59 μg/ml and 109.68 μg/ml, respectively. Compared to the control group, thiopental at ≥25 μg/ml and propofol at ≥50 μg/ml exhibited cytotoxicity. Additionally, propofol exhibited significantly lower cytotoxic effects than thiopental did.

Conclusion: Our study showed that both propofol and thiopental exerted significant cytotoxic effects on HEK-293 cells at concentrations exceeding clinical levels, primarily by increasing intracellular ROS levels and inducing apoptosis. Future research in this area will deepen our understanding of these mechanisms and improve patient safety in clinical anesthesia practice.

## Introduction

Propofol and thiopental are sedative and hypnotic agents widely used in anesthesia practice worldwide, with proven safety and efficacy. They play critical roles in a wide range of patients from neonates to the elderly, such as anesthesia induction, sedation, analgesia, and antioxidant therapy [1,2]. These drugs are used as infusions, especially for total intravenous anesthesia (TIVA) and long-term sedation in intensive care units. However, long-term and high-dose use may lead to accumulation in plasma and tissues, resulting in high drug concentrations, which increases the potential risk of toxicity (e.g., nephrotoxicity) [3,4].

These agents, which are generally considered safe at standard clinical doses, have recently been the subject of increasing concerns about their possible toxic effects at the cellular and molecular levels. There is increasing evidence that toxic effects, especially anesthesia-related neurotoxicity, may develop, making the pediatric patient group particularly vulnerable. Studies on children have indicated that drugs such as propofol and thiopental can damage neurological development, and it has been emphasized that more serious effects may occur with long-term use [1,2]. Therefore, careful monitoring and dose management are required, especially during high-dose or long-term administration. This situation shows that propofol and thiopental should be used not only by considering their clinical benefits but also by considering their possible long-term side effects. In clinical practice, more research and a careful monitoring protocol are required to ensure patient safety during the use of these agents, especially in long-term sedation infusions.

Both propofol and thiopental have positive pharmacokinetic profiles characterized by short durations of action and rapid clearance rates, making them ideal for long-term sedation in intensive care units and for the induction and maintenance of TIVA under anesthesia [3,4]. Propofol and thiopental plasma concentrations at clinical doses have been reported to be 11 μg/ml and 41.6 μg/ml, respectively [5,6]. The fat solubility of these drugs may cause high concentrations and accumulation at plasma and tissue levels. In addition, their dose-dependent effects may lead to systemic toxicity, and possible side effects such as fluctuations in blood pressure, heart rate abnormalities, and pain at the injection site, especially during propofol administration, may occur [3,7,8]. Although the mechanisms underlying these effects are not yet fully understood, they are thought to involve the modulation of gamma-aminobutyric acid (GABA)-mediated chloride channels in the central nervous system [9,10].

Propofol is primarily metabolized by the liver (60%) but also undergoes significant extrahepatic metabolism (40%), and the kidneys play an important role in its clearance [11]. Prolonged infusions may lead to drug accumulation in tissues and blood, which may delay emergence from anesthesia [12]. In contrast, the metabolism and clearance of thiopental are largely independent of hepatic blood flow, with a low clearance rate (0.21 L/min) predisposing it to accumulation in tissues, especially during prolonged or repeated administration [10]. The drug's metabolites are excreted via the kidneys in the form of glucuronidates, with a highly variable elimination half-life ranging from five to 22 hours [10], further complicating its safe use in long-term therapy.

Propofol and thiopental have remarkable antioxidant properties, as shown in animal models and in vitro studies [4,13]. For example, Zheng et al. found that propofol acts as an effective agent in reducing sepsis-induced kidney injury in vivo and in vitro, whereas Song et al. showed that propofol protects human kidney proximal tubular cells (HK-2) from oxidative damage induced by hydrogen peroxide (H2O2) [14,15]. However, other studies have reported that propofol may increase oxidative stress in certain organs and tissues by increasing reactive oxygen species (ROS) levels and decreasing antioxidant reserves [16,17].

Propofol and thiopental are known to exhibit cytotoxicity at doses higher than the clinical dose, and studies have shown that these drugs can induce apoptosis and necrosis through the excessive production of ROS [18,19]. Although no toxic effects have been observed at standard clinical doses, high, repeated, and infusion (e.g., TIVA, sedation, etc.) doses can disrupt the delicate balance between the oxidant and antioxidant systems, triggering oxidative stress, cell damage, and apoptosis [10,13,17,18,20]. Because of their high metabolic activity and constant exposure to circulating toxins, the kidneys are particularly vulnerable to the nephrotoxic effects of anesthetic agents, making them an important focus for studying drug-induced cytotoxicity [21]. Human renal epithelial cells (HEK-293) serve as an optimal in vitro model to investigate renal cell physiology [15,22]. Therefore, we used human renal epithelial cells (HEK-293) in our study.

Kidneys play a role in drug metabolism via the renal enzyme system. In particular, they cause the biotransformation of drugs and toxins via renal cytochrome P450 (CYP450) enzymes and flavin-containing monooxygenases. This further emphasizes the importance of investigating nephrotoxicity in the context of anesthesia [23]. These pathways can produce ROS and nephrotoxic metabolites that contribute to oxidative stress and lead to DNA damage, lipid peroxidation, and protein degradation [21]. Consequently, understanding the mechanisms of drug-induced nephrotoxicity is essential for optimizing the safety and efficacy of anesthetic regimens.

Although studies have demonstrated the toxic and protective effects of propofol and thiopental, the intracellular mechanisms of action that drive these results remain unclear. With this in vitro study, we aimed to provide new insights into the potential cytotoxic effects of these anesthetic agents, which are widely used at concentrations exceeding clinical doses, using HEK-293 renal epithelial cells as a model system. Our study aimed to contribute to the growing evidence that anesthetic drugs may have undesirable cytotoxic effects at doses higher than clinical doses by investigating their effects on cytotoxicity, apoptosis, and ROS production.

## Materials and methods

Cells and culture conditions

In this study, we obtained and stocked cells from the American Type Culture Collection (ATCC); HEK-293 cells (human kidney epithelial cell, ATCC code: CRL-1573 CD BioSciences, USA) were used [22]. Our study followed the Checklist for Reporting In Vitro Studies (CRIS) guidelines [24] (see Appendix A). Cell lines were grown in Dulbecco's Modified Eagle Medium: F12 (DMEM) medium containing 1% P/S, 10% fetal bovine serum (FBS), and 1% glutamine. All the cells were incubated at 37°C in an atmosphere of 5% CO. Cells were removed with a mixture of 0.25% trypsin and 0.03% ethylenediaminetetraacetic acid (EDTA) and passaged at a ratio of 1:2 or 1:3, as recommended by ATCC, with unused cells containing 95% broth and 5% dimethyl sulfoxide (DMSO). The prepared cell freezing solution was first stored in a deep freezer at -80°C for a short or long term in liquid nitrogen.

Cytotoxicity analysis with 3-(4,5-dimethylthiazol-2-yl)-2,5-diphenyltetrazolium bromide (MTT)

The medium was refreshed 24 hours after HEK-293 cells were seeded in 96 sterile plates at 104 cells per well. The concentration range for the drugs applied to the cells was carefully selected, spanning 10 doses (2.5, 5, 10, 25, 50, 100, 200, 250, 400, and 500 µg/ml), to comprehensively evaluate the dose-dependent effects. Propofol and thiopental drugs were administered in 10 doses with three repetitions. No drugs were administered in the control group. The cell lines were incubated in an incubator for 24 hours after drug administration. After drug administration, the cell medium was removed. The cytotoxic effects of propofol and thiopental were evaluated using the MTT method. Absorbance was read at 570-690 nm using a plate reader (Multiskan GO, Thermo Fisher Scientific, Waltham, MA, USA) [25]. Graphics were then created (see Appendix B). The half-maximal inhibitory concentrations (IC_50_) were then calculated. According to the IC_50_ values found in the cytotoxicity tests, other tests were performed for propofol and thiopental at doses of 100 and 200 μg/ml.

Cell morphology and acridine orange/ethidium bromide (AO/EB) analysis 

Images of cell morphology were obtained using an inverted microscope (Olympus CKX, Olympus Life Science, Tokyo, Japan) at 20× magnification. Apoptosis fluorescence in the cells was examined using the AO/EB staining method. After 24 hours, the medium was removed, 50 μL of AO/EB dye (Sigma-Aldrich, St. Louis, MO, USA) was added, and images were taken at 20× magnification using a fluorescence microscope (Olympus CKX 51, DP73).

Determination of apoptosis by annexin V/propidium iodide (PI) method

This analysis was performed using a commercial fluorescein isothiocyanate (FITC) Annexin V Apoptosis Detection Kit I (BD Biosciences, Franklin Lakes, NJ, USA) according to the manufacturer's instructions. The cells were lifted using trypsin and transferred to Eppendorf tubes. Then, 5 μL of fluorochrome-conjugated annexin V and 5 μL of PI dye were added. One hundred microliters of 1× binding buffer was added to the cells, centrifuged at 1200 rpm, and incubated for five minutes. Finally, the cells were analyzed using flow cytometry. Annexin V is shown in green and PI in red. Living cells were differentiated into ((FITC−)/(PI−)), early and moderately apoptotic ((FITC+)/(PI−)), and late apoptotic and necrotic cells ((FITC+)/(PI+)).

Intracellular ROS determination

Intracellular free radical exchange was performed according to the protocol of a commercially available kit (MHC100111, Merck Millipore, Burlington, MA, USA). The Muse® Oxidative Stress Kit (Merck Millipore, Burlington, MA, USA) provides quantitative (cell count and percentage) measurements of ROS, that is, superoxide radicals, in cells exposed to oxidative stress. After drug administration, cells were harvested using trypsin and washed with cold phosphate-buffered saline (PBS). After adding 100 µL of the ROS working solution, the cells were incubated at 37°C for 30 minutes. After incubation, the cells were analyzed using flow cytometry (FACSVia, BD Biosciences).

Statistical analysis

The data used in the study were analyzed using IBM SPSS Statistics for Windows, V. 24.0 (IBM Corp., Armonk, NY, USA). Data are presented as mean ± standard deviation (SD). The normality of the data was tested using the Shapiro-Wilk test. For the comparisons of groups, Student's t-test was used for pairs and the ANOVA test was used for double groups. P < 0.05 was regarded as statistical significance.

## Results

Cytotoxic effects of propofol and thiopental on HEK-293 cells

**Figure 1:**
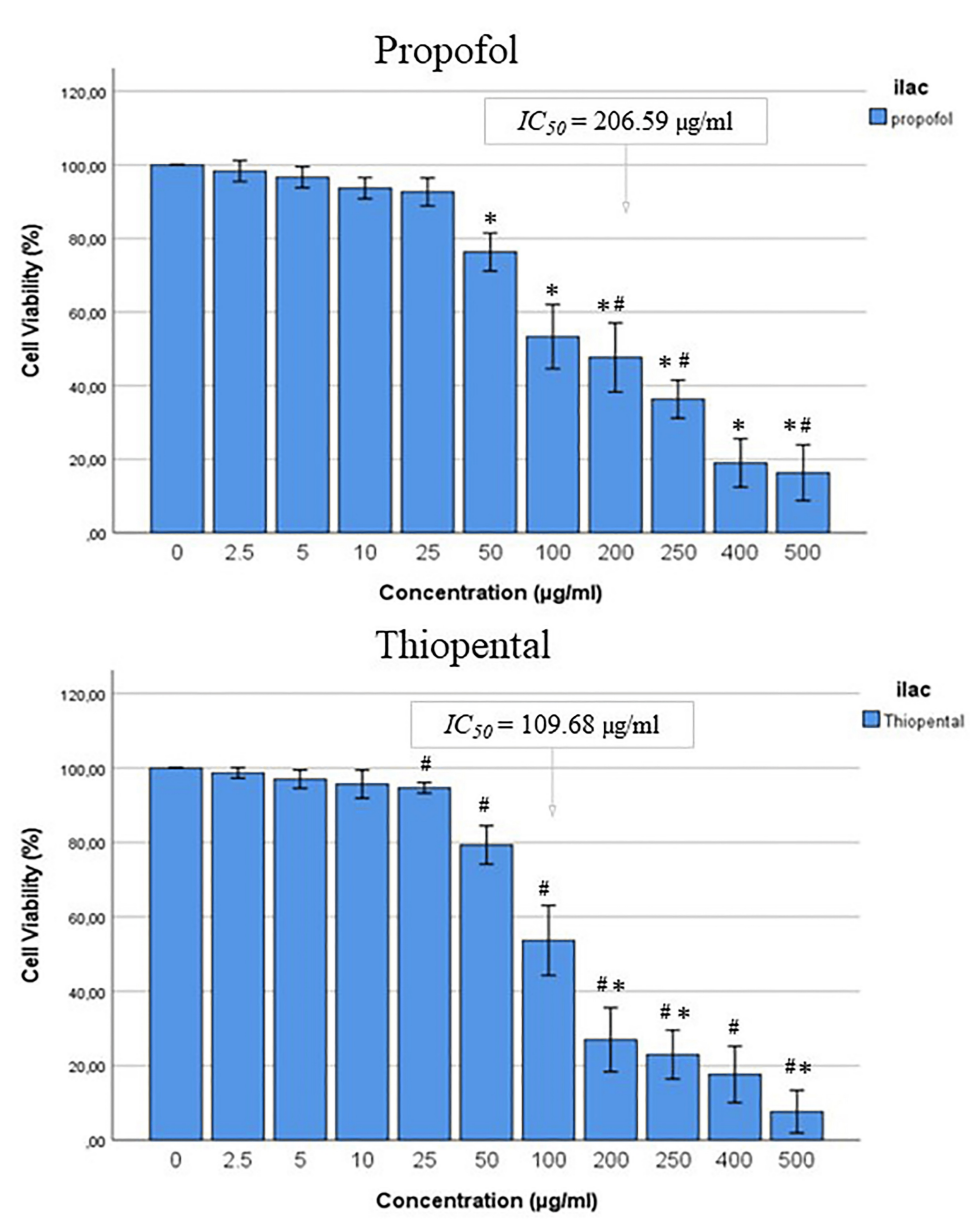
% changes in the viability of HEK-293 cells treated with different concentrations of propofol and thiopental for 24 hours The data obtained are shown as mean ± standard deviation. ^*^P < 0.001 vs control group for propofol. ^#^P < 0.001 vs control group for thiopental. ^*#^P < 0.001 vs thiopental group for propofol. IC_^50^_: half-maximal inhibitory concentration

The analysis revealed a dose-dependent increase in the cytotoxic effects of both drugs (Figure 1). The IC_50_ values in HEK-293 cells were 206.59 μg/ml for propofol and 109.68 μg/ml for thiopental. Compared with the control group, concentrations of thiopental ≥25 μg/ml and propofol ≥50 μg/ml exhibited significant cytotoxicity (P < 0.001). When propofol and thiopental were compared using equivalent doses, propofol demonstrated significantly lower cytotoxicity than thiopental at doses of 200 μg/ml, 250 μg/ml, and 500 μg/ml (P < 0.001).

Effects of propofol and thiopental on HEK-293 cell morphology

**Figure 2:**
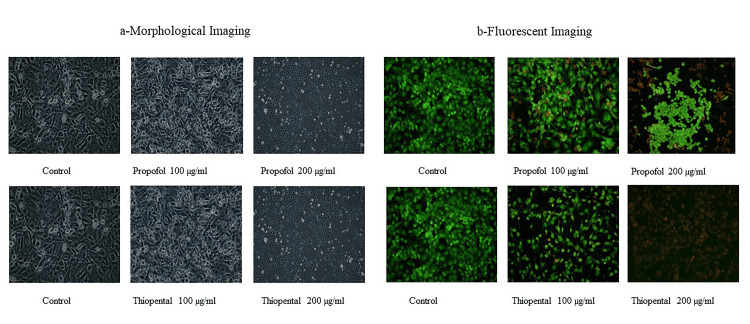
Morphological (a) and AO/EB fluorescence (b) staining on HEK-293 cells at 20× magnification Live cells in green, apoptotic cells in orange, and necrotic cells in red. AO/EB: acridine orange/ethidium bromide

When the effects on cell morphology were compared with the control group, a decrease in the number of living cells and an increase in the number of apoptotic cells were observed as the dose increased for both drugs, although they were more pronounced in the thiopental group (Figure 2a). The apoptotic effect of the drugs on HEK-293 cells was further examined by AO/EB fluorescent staining (Figure 2b). In the obtained images, viable cells appear green, apoptotic cells appear orange, and necrotic cells appear red. Comparisons of the apoptotic effects of the drugs revealed that thiopental induced greater apoptosis than propofol and greater apoptotic and cytotoxic effects were observed with thiopental.

Flow cytometric annexin V analysis of the apoptotic effects of propofol and thiopental on HEK-293 cells

**Figure 3:**
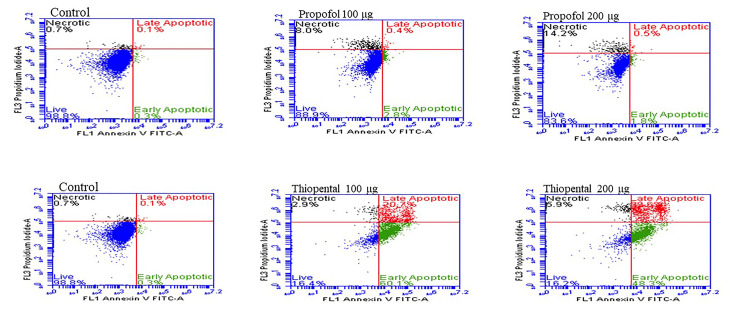
Flow cytometric annexin V analysis of the apoptotic effects of drugs on HEK-293 cells FITC: fluorescein isothiocyanate

**Figure 4:**
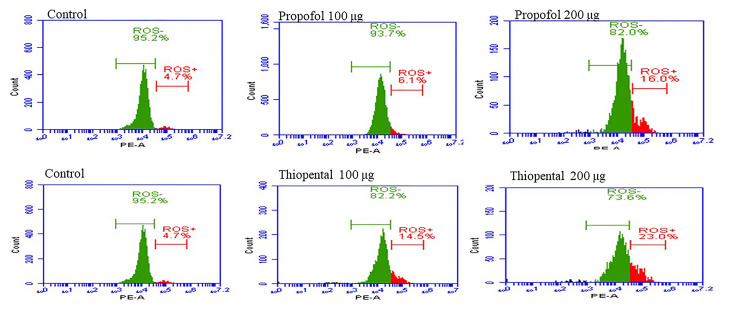
Flow cytometric investigation of the effects of propofol and thiopental on intracellular free radical (ROS) formation of HEK-293 cells ROS: reactive oxygen species

At doses of 100 µg/ml and 200 µg/ml, 71.93% and 66.50% of HEK-293 cells treated with propofol remained viable, respectively, whereas 4.03% and 3.86% underwent late apoptosis (Table 1, Figure 3). In contrast, 58.16% and 51.56% of the thiopental-treated HEK-293 cells remained viable at the same doses; 12.46% and 3.40% underwent late apoptosis, respectively (Table 1, Figure 4). These findings represent statistically significant differences compared with the control group. Furthermore, comparisons between the drugs revealed statistically significant differences in viability, late apoptosis, and necrosis at 200 µg/ml (P < 0.005).

**Table 1:** Flow cytometric annexin V analysis of the apoptotic effects of drugs on HEK-293 cells Values are given as mean ± standard deviation. P = 0.001 indicates a comparison of propofol and thiopental groups for 100 µg/ml doses. ^*,#,¥,α ^indicate P-values less than P < 0.001 for comparison of groups, propofol, thiopental, and control for all doses. ^€ ^indicates independent samples t-test.

Viability	Control	Doses (µg/ml)	Propofol	Thiopental	t-statistic	P-value^€^
Live	97.10 ± 1.86	100 µg/ml	71.93 ± 2.33	58.16 ± 1.66	-8.32	=0.001^*^
		200 µg/ml	66.50 ± 1.41	51.56 ± 1.90	-10.92	<0.001^*^
Early apoptotic	0.63 ± 0.41	100 µg/ml	0.23 ± 0.15	0.33 ± 0.15	0.80	=0.468^#^
		200 µg/ml	0.36 ± 0.11	0.63 ± 0.15	2.41	=0.073^#^
Late apoptotic	0.26 ± 0.20	100 µg/ml	4.03 ± 1.00	12.46 ± 1.22	9.23	=0.001^¥^
		200 µg/ml	3.86 ± 0.56	3.40 ± 0.62	-0.96	=0.393^¥^
Necrotic	1.93 ± 1.88	100 µg/ml	23.80 ± 2.78	29.00 ± 2.52	2.40	=0.075^α^
		200 µg/ml	29.23 ± 1.15	43.06 ± 3.21	7.02	=0.002^α^

Flow cytometric analysis of the effects of propofol and thiopental on intracellular ROS formation in HEK-293 cells

We found that the intracellular free radical levels increased in a dose-dependent manner (Table 2, Figure 4). At a dose of 100 μg, propofol resulted in 21.06% intracellular free radicals, whereas thiopental resulted in 18.36% intracellular free radicals. At 200 μg, propofol and thiopental exhibited intracellular free radical levels of 26.13% and 39.16%, respectively (P = 0.001). ROS generation and cell death were induced at lower doses of propofol compared to thiopental.

**Table 2:** ROS effect of different doses of drugs on HEK-293 cells Values are given as mean ± standard deviation. P = 0.001 indicates a comparison of propofol and thiopental groups for 100 µg/ml doses. ^*,#^ indicate P-values less than P < 0.001 for comparison of groups, propofol, thiopental, and control for all doses. ^€^ indicates independent samples t-test. ROS: reactive oxygen species

	Doses	Control	Propofol	Thiopental	t-statistic	P-value^€^
ROS (−)	100 µg/ml	98.30 ± 1.25	79.36 ± 3.44	82.70 ± 0.36	1.69	=0.171^*^
	200 µg/ml	95.93 ± 1.90	74.30 ± 1.85	61.30 ± 0.81	-11.12	<0.001^*^
ROS (+)	100 µg/ml	4.03 ± 1.89	21.06 ± 3.30	18.36 ± 0.45	-1.40	=0.234^#^
	200 µg/ml	4.03 ± 1.89	26.13 ± 1.87	39.16 ± 1.45	9.52	=0.001^#^

Overall, increased doses of propofol and thiopental led to decreased cell viability and increased ROS formation. Both drugs exhibited statistically significant differences compared with the control group (P < 0.001).

## Discussion

When comparing the effects of propofol and thiopental on HEK-293 cells at two different high doses, we observed that both agents induced an increase in intracellular ROS levels, triggering apoptosis and cell death. The IC_50_ values for propofol and thiopental were 206.59 μg/ml and 109.68 μg/ml, respectively. This indicates that thiopental exhibits significantly higher cytotoxicity than propofol at equivalent high doses. These findings suggest that although both drugs exhibit minimal cytotoxicity at standard clinical concentrations, propofol may be a safer option when higher doses are required in clinical practice (e.g., prolonged sedation infusion in the intensive care unit).

Propofol and thiopental are widely used in anesthesia and sedation, with established safety profiles at recommended clinical doses [3,4]. However, prolonged use or administration of high doses can lead to rare but severe complications [10,26,27]. The fat solubility of these drugs may cause high concentrations and accumulation at plasma and tissue levels. In particular, in previous studies, propofol plasma levels were found to be 36.1 ± 8.2 μg/ml at clinical doses, suggesting that high plasma levels may develop with long-term infusions [5,13]. These findings suggest that the propofol doses used in this study (100-200 μg/ml) can be achieved through clinical infusions. Consistent with our results, Li et al. reported that propofol exhibited minimal cytotoxicity at doses up to 100 μM in in vitro models [5]. A comparison of the two agents at equivalent high doses showed that thiopental showed significantly higher cytotoxicity (indirectly nephrotoxicity) than propofol, suggesting that caution should be exercised in the use of thiopental in high-dose clinical applications.

Several studies have explored the protective and potential therapeutic roles of propofol, particularly in models of renal injury. For instance, Echavarría et al. demonstrated that preconditioning with propofol (7.5 mg/kg) one hour prior to ischemia reduced the markers of kidney injury and improved renal function following ischemia-reperfusion injury [28]. Zheng et al. speculated that propofol could serve as an effective therapeutic agent for suppressing sepsis-induced kidney injury in vivo and in vitro [14]. Song et al. suggested that propofol protects HK-2 human kidney proximal tubular epithelial cells from oxidative stress caused by H2O2 in vitro and has antioxidant potential [15]. On the other hand, thiopental has less antioxidant activity, and some reports indicate that thiopental increases oxidative stress by increasing oxidant levels while depleting antioxidants in organ tissues [4,29].

Despite their protective properties in certain tissues, both propofol and thiopental have been associated with cell death by apoptosis and necrosis, especially when administered in high or repeated doses [10,13,17-19,27]. Sumi et al. showed that propofol at clinically relevant concentrations suppressed mitochondrial function and induced apoptosis and necrosis in human neuroblastoma cells through ROS production and metabolic reprogramming [18]. Similar findings have been observed in other cell types, including human promyelocytic leukemia cells, where high doses of propofol impaired oxygen utilization and triggered apoptosis [16,30]. Thiopental has also been associated with increased ROS and oxidative damage in human lymphocytes and liver tissues [19].

In our study, although propofol and thiopental had no cytotoxic effects at clinical doses, both agents caused significant apoptosis, necrosis, and increased intracellular ROS levels at higher doses. Importantly, when we compared the two drugs at equimolar concentrations, thiopental showed greater toxicity, leading to more pronounced cytotoxicity, necrosis, and apoptosis. This finding highlights the need for careful dose management and biochemical monitoring of patients with normal or borderline renal function, especially in clinical situations where high doses of thiopental may be required (e.g., during prolonged sedation infusion in the intensive care unit). In such patients, it is critical that drug levels be closely monitored and alternative sedation strategies be considered when necessary, to prevent renal injury and minimize potential toxic effects.

The exact mechanisms by which propofol and thiopental exert their effects remain largely unknown. Both agents have been shown to modulate GABA-mediated chloride channels in the brain, but their specific interactions with kidney cells and other organ systems are unclear [3,4]. Given the critical role of kidneys in anesthetic metabolism and excretion, HEK-293 cells serve as an appropriate model for studying the nephrotoxic effects of these drugs [11,15,22]. By using HEK-293 cells, we aimed to elucidate the clinical and high-dose effects of propofol and thiopental on renal cell physiology, with a focus on cytotoxicity, apoptosis, and ROS generation.

As anesthesia techniques continue to evolve, it is vital to ensure the safe administration of anesthetic agents, especially in patients requiring prolonged sedation and infusions of anesthesia in intensive care settings. When used for prolonged sedation in intensive care units, exposure to oxidative or antioxidant stress before, during, and after surgery and during recovery is a significant concern for these patients [4]. Anesthetists should take proactive measures to minimize oxidative stress and improve patient outcomes, particularly in high-risk populations. However, further studies are needed to enhance our understanding of how anesthetic agents influence oxidative stress and clinical outcomes, enabling the development of strategies to mitigate these risks.

This study had several limitations. First, we used cultured HEK-293 cells rather than primary cells from kidney tissues, which may have limited the generalizability of our findings to in vivo conditions. Second, although we used 10% FBS in our cell culture experiments, we were unable to determine the free drug fractions, which may have influenced the observed cytotoxicity. Another limitation of our study is that the results from this cell model could not be directly applied in clinical settings.

Future research should focus on translating these in vitro findings into in vivo models to better understand the systemic implications of prolonged or high-dose anesthetic use. Further investigation into the mechanisms underlying ROS generation and oxidative stress in response to these anesthetics could lead to the development of protective strategies aimed at minimizing their adverse effects. Identifying biomarkers that can predict susceptibility to anesthetic-induced oxidative stress will be crucial for tailoring anesthesia protocols to reduce potential complications.

## Conclusions

Our study shows that both propofol and thiopental exert significant cytotoxic effects on HEK-293 cells at concentrations exceeding the clinical levels, primarily by increasing intracellular ROS levels and inducing apoptosis. Clinicians should be careful to monitor drug levels and be aware of the potential for oxidative stress-induced damage in patients exposed to prolonged- or high-dose anesthesia. Future research in this area will deepen our understanding of these mechanisms and improve patient safety in clinical anesthesia practice.

## Figures and Tables

**Figure 5:**
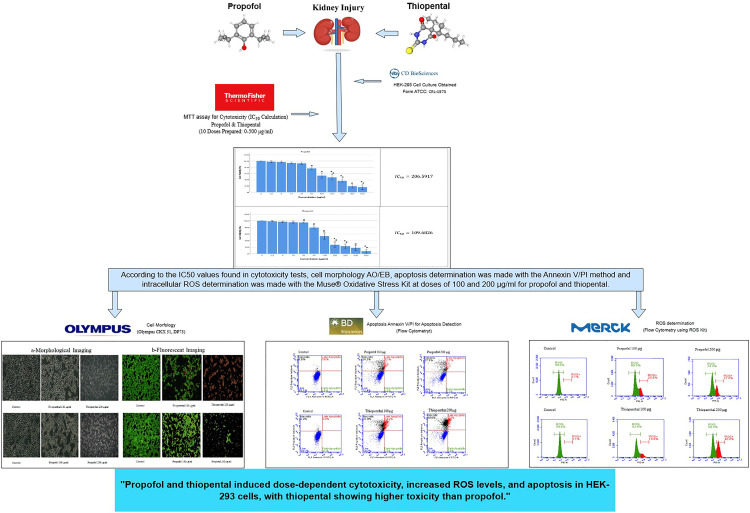
Graphical abstracts IC_50_: half-maximal inhibitory concentration; AO/EB: acridine orange/ethidium bromide; ROS: reactive oxygen species; MTT: 3-(4,5-dimethylthiazol-2-yl)-2,5-diphenyltetrazolium bromide; ATCC: American Type Culture Collection

## Appendices

Appendix A

**Table 3:** CRIS guidelines* *This checklist was developed by the authors based on the following sources: [31,32] CRIS: Checklist for Reporting In-vitro Studies www.consort-statement.org

Section/topic	Item no.	Checklist item	Reported on page no.
Title and abstract			
	1a	Identification as an in vitro/laboratory study in the title	1
	1b	Structured summary of trial design, methods, results, and conclusions	2
Introduction			
Background and objectives	2a	Scientific background and explanation of the rationale	2-4
	2b	Specific objectives or hypotheses	4
Methods			
Interventions	3	The intervention for each group, including how and when they were actually administered, with sufficient detail to allow replication	5-6
Outcomes	4	Completely defined pre-specified primary and secondary outcome measures, including how and when they were assessed	5-6
Sample size	5	How sample size was determined	NA
Randomisation			
Sequence generation	6	Method used to generate the random allocation sequence	NA
Allocation concealment	7	Explain the mechanism used to implement the randomisation sequence (such as sequentially numbered containers) and the steps taken to conceal the sequence until interventions were assigned. NA (not applicable) (randomisation is not applicable because the nature of the study is an experimental cell study)	NA
Implementation	8	Who generated the random allocation sequence, who enrolled teeth, and who assigned teeth to intervention. NA (not applicable) (random allocation cannot be applied because the nature of the study is an experimental cell study)	NA
Blinding	9	If done, who was blinded after assignment to interventions (for example, care providers, those assessing outcomes) and how	NA
Statistical methods	10	Statistical methods used to compare groups for primary and secondary outcomes	6
Results			
Numbers analysed	11a	For each group, the number of "items" (drugs) included in each analysis and whether the analysis was by the original assigned groups	6-7
Outcomes and estimation	11b	For each primary and secondary outcome, results for each group and the estimated effect size and its precision (such as 95% confidence interval)	NA
Discussion			
Limitations	12a	Trial limitations, addressing sources of potential bias, imprecision, and, if relevant, multiplicity of analyses	10
Generalisability	12b	Generalisability (external validity, applicability) of the trial findings	10
Interpretation	12c	Interpretation consistent with results, balancing benefits and harms, and considering other relevant evidence	10
Other information			
Protocol	24	Where the full trial protocol can be accessed, if available	NA
Funding	25	Sources of funding and other support (such as the supply of drugs), role of funders	11

Appendix B
